# Educational Needs for Coaching Judo in Older Adults: The EdJCO Focus Groups

**DOI:** 10.3390/sports11080143

**Published:** 2023-07-31

**Authors:** Federico Palumbo, Simone Ciaccioni, Flavia Guidotti, Roberta Forte, Envic Galea, Attilio Sacripanti, Nuša Lampe, Špela Lampe, Toma Jelušić, Slaviŝa Bradić, Maria-Loredana Lascau, Alina Rodica-Borza, Raúl Camacho Pérez, Fernando Diéguez Rodríguez-Montero, Mesut Kapan, Kaya Gezeker, Laura Capranica, Antonio Tessitore

**Affiliations:** 1 Department of Movement, Human and Health Sciences, Italian University of Sport and Movement “Foro Italico”, 00135 Rome, Italy; federico.palumbo90@gmail.com (F.P.); simoneciaccioni@yahoo.it (S.C.); roberta.forte@uniroma4.it (R.F.); laura.capranica@uniroma4.it (L.C.); antonio.tessitore@uniroma4.it (A.T.); 2 International Judo Federation Academy Foundation, XBX 1421 Ta’ Xbiex, Malta; envic.galea@ijf.org (E.G.); attilio.sacripanti@uniroma2.it (A.S.); 3 Judo Club Golovec, 1000 Ljubljana, Slovenia; nusa@judogolovec.si (N.L.); spela.lampe@judogolovec.si (Š.L.); 4 Zajednica Sportskih Udruga Grada Rijeke “Riječki Sportski Savez”, 51000 Rijeka, Croatia; toma@rss.hr (T.J.); slavisa.bradic@rss.hr (S.B.); 5 Judo Club Liberty Oradea, 410437 Oradea, Romania; loredana.maria.lascau@gmail.com (M.-L.L.); alina81gym@gmail.com (A.R.-B.); 6 Club de Judo Newton, 28609 Sevilla La Nueva, Spain; raukamp@hotmail.com (R.C.P.); fdieguez1@hotmail.es (F.D.R.-M.); 7 Izmir Alsancak Gymnastics Specialized Sports Club, İzmir 35210, Türkiye; mesutkapan@hotmail.com (M.K.); kayagezeker@hotmail.com (K.G.)

**Keywords:** judo, martial arts, older individuals, coaches, successful aging, focus groups

## Abstract

Judo coaches are urged to develop specific competencies and skills for addressing the special needs of older practitioners. Thus, the purpose of this study was to investigate the experts’ opinions on judo training in late adulthood to develop sound educational programs for coaches of older judo practitioners. Overall, eighty-eight experts from an international consortium of judo and educational partners participated in national focus groups. During the focus groups, experts discussed five themes and generated statements pertinent to educate coaches to support older judo practitioners (e.g., benefits; necessary knowledge; risks; training groups definition; tools; and tests for monitoring training plans). The initial list of 262 statements was synthesized, validated, analyzed, and organized into a final list of 55 statements and six macro-areas: aging process (*n* = 10); safety and first aid (*n* = 6); physiology and fitness (*n* = 12); psychology and mental health (*n* = 11); organization and environment (*n* = 5); adapted judo teaching and training (*n* = 11). The present international eminence-based study, harmonizing diverse intercultural perspectives, highlighted the specific needs of older judo practitioners. The results of this study will contribute to the structure of a sound educational program for coaches of older judo practitioners to enhance the quality of older adults’ sports experiences by linking safety, enjoyment, social interactions, and learning principles.

## 1. Introduction

Healthy aging has become a high priority in contemporary society, also considering the relevant and continuous increase of the older adults’ share in the general population [1]. Thus, the development and implementation of specific policies addressing the promotion of health-enhancing active lifestyles for older people represent a strategic action worldwide [2,3,4,5]. To address the loss of physiological, functional, and cognitive capacities naturally accompanying the aging process, specific physical activity interventions in late adulthood play a crucial role in delaying age-related decline, counteracting the onset of chronic diseases, and reducing the mortality risk associated with sedentary behaviors [6,7]. In fact, active lifestyles have been associated with behavioral, physiological, psychological, and social well-being, improving functional abilities, and preventing the occurrence of falls in older individuals [8,9,10,11,12]. In this framework, older individuals are recommended to engage >2 days weekly in a multi-component physical activity, encompassing both aerobic exercise and resistance training involving major muscle groups, complemented by the combination of exercise and cognition (e.g., dual tasks) [4,13,14]. Furthermore, physically active older individuals are considered good examples of successful aging, and the involvement of older people in sport-based programs resulted in particularly effective in preserving their health with advancing years [15,16,17,18,19]. 

Judo (jū = gentle; dō = way) is a martial art and an Olympic combat sport, but more a means of education and a method of personal and social improvement through the execution of various techniques, according to its founder, the Japanese educator Jigoro Kano Sensei. Due to its inner characteristics stimulating coordination, dynamic balance, bone health, and mental control, judo has been considered particularly suitable for older people [11,16,20,21,22]. Based on the principles of “maximum efficient use of mind and body” and “mutual welfare and benefit” [23], judo encompasses self-defense techniques implemented in group contexts, which improve self-control, discipline, active lifestyles, physical and mental health, and a special group of techniques allowing to fall in a controlled and safe way (i.e., ukemi) [10,11,23,24]. 

Coaches’ preparedness is a crucial component for structuring and monitoring safe and effective sports programs for older individuals [25]. In this respect, coaches are urged to develop specific competencies and skills for addressing the special needs of older practitioners and lifelong sports involvement [25,26,27]. To harmonize the judo certification worldwide, the International Judo Federation Academy Foundation delivers standardized judo educational courses for coaching certifications, mainly focused on youth athletes and elite athletes, with no information on older practitioners provided ((https://academy.ijf.org/ (accessed on 19 July 2023)). To gain a comprehensive understanding of the benefits of judo in older adults and to assist coaches in effectively promoting judo training in healthy settings, the European Commission recently funded the “EDucating Judo Coaches for Older practitioners (EdJCO)” Project (622155-EPP-1-2020-1-IT-SPO-SCP) under the Erasmus+ Sport Programme [28,29]. The EdJCO project includes a consortium of sports (i.e., judo clubs from Croatia, Romania, Slovenia, Spain, Türkiye) and educational partners (i.e., the University of Rome Foro Italico, Italy, and International Judo Federation Academy Foundation, Malta) from seven countries. In particular, the consortium includes judo clubs holding the required European ERASMUS+ Participant Identification Code (PIC), encompassing members with long-term cooperation with the International Judo Federation as educators, coaches, referees, and scientific experts. The main purpose of the EdJCO project is to promote evidence- and eminence-based knowledge on judo training for older judo practitioners and to develop recommendations for addressing coaches’ educational needs through the implementation of an educational program targeting judo practice in late adulthood. Regarding evidence-based knowledge, a systematic literature review on the methodological approaches of teaching judo techniques to middle-aged and older practitioners highlighted the need for specific knowledge on the main aspects to be considered when constructing training programs for coaching older judo practitioners [11].

Therefore, the purpose of the present eminence-based study was to explore experts’ perceptions regarding the knowledge and competencies that judo coaches should acquire to plan safe judo training for older individuals. In particular, the focus group methodology was deemed appropriate to explore the opinions of academic and judo experts on the necessary knowledge to be provided to coaches on the benefits, the risks, and the adapted judo methodologies for implementing effective and safe programs for novice and expert older judo practitioners [30]. It was hypothesized that the main outcomes of this investigation would provide relevant information to develop recommendations for academic institutions and judo bodies for structuring sound educational programs for coaches of older judo practitioners.

## 2. Methods

### 2.1. Study Design

The present study was approved by the European Commission (622155-EPP-1-2020-1-IT-SPO-SCP) and the Institutional Review Board of the University of Rome Foro Italico (CAR73/2021). An ethnographic research approach was deemed appropriate to construct an international educational programme for judo coaches encompassing educational units and intelligible content according to the needs of coaches from different social and cultural settings [31]. To gather a variety of information and different perspectives for a comprehensive understanding of key educational aspects of judo, the EdJCO team reached a consensus on a purposeful recruitment and inclusion criteria of a variety of scholars (university professors and researchers expert in biomechanics, communication, education, kinesiology, nutrition, physiology, and psychology), medical doctors (with a specialization in geriatrics, family medicine, and sports medicine), and experts of judo (>second DAN with coaching expertise >5 years) and other combat sports in the seven project’s participating countries (i.e., Croatia, Italy, Malta, Romania, Slovenia, Spain, Türkiye). Furthermore, the use of the focus group methodology was deemed suitable to provide the participants with plenty of possibilities to freely interact and discuss specific themes of judo for older individuals and to agree on the key issues to be included in an educational programme for coaches [30,32]. With participants playing a central role within the group discussion and the researcher/observer proposing, facilitating, and moderating a logical sequence of open-ended questions on specific topics, the focus groups proved to be an effective and sustainable qualitative research methodology [9,30,33,34,35,36]. To ensure the relevance and accuracy of the outcomes, the EdJCO project team produced guidelines for the conduction of the focus groups, encompassing the information to be provided to participants before the group discussion, the methodological approach to stimulate balanced participation, the data collection and synthesis, and the translation and back-translation of statements from the national languages of the EdJCO project partners (e.g., Croatian, Italian, Romanian, Slovenian, Spanish, and Türkish) to English (Supplementary Material S1). 

Following the findings of a systematic literature review [11], the research team engaged in a thorough discussion and identified five main themes to gather the experts’ opinions and insights regarding the benefits and risks of practicing judo, the practical knowledge for coaching older practitioners, and the training monitoring tools to assess and control the training load in the late adulthood were sought through five open-ended questions [37]:

1. What are the most relevant benefits judo coaches should be aware of when training former or novice older judo practitioners?
2. What are the most relevant information judo coaches should be aware of when training novice older judo practitioners?
3. What are the most relevant information judo coaches should be aware of to prevent/manage risks when training former or novice older judo practitioners?
4. Which are the main criteria for defining judo training groups in relation to participants’ judo expertise, chronological/functional age, and/or sex?
5. What are the most relevant tools/tests/measurements for monitoring training plans for former or novice older judo practitioners?

Overall, this study met the eight criteria for qualitative excellence [37,38]: (1) worthy topic, grounded on research on judo practice in the late adulthood [11,16]; (2) stringent rigor, guaranteed by the EdJCO guidelines for national focus groups with experts, including clear definition of the research questions, the purposeful selection of participants, the instructions to be provided to focus groups participants, the focus group standard operating procedures, and the data collection, synthesis, and translation; (3) sincerity, attained through a co-construction process between the members of the research team leading the focus groups and the experts’ free discussion upon the proposed themes without interferences, and the impartial analysis of the collected data; (4) credibility, highlighted in the outcomes of the focus groups presenting different ideas, opinions, and statements; (5) resonance, based on the participation of judo coaches of older practitioners, which intrinsically enhance the relevance of the results; (6) significant contribution of the present findings, which complement the evidence-based knowledge [11] to enhance the awareness of coaches, sports scientists, and sport bodies regarding the specificity of judo training for older individuals, the need to develop an adequate training programme for judo coaches and future research in the field; (7) ethical compliance of the study conducted in conformity with the Declaration of Helsinki, substantiated by external (i.e., the European Commission financing the EdJCO project) and internal (i.e., the Institutional Review Board of the University of Rome “Foro Italico”) approvals, which included the organization of national focus groups in seven European countries; and (8) meaningful coherence, attained through the experts’ opinions on the coherence between the research aims, procedures, outcomes, and interpretation.

### 2.2. Participants

According to the literature [39] and in agreement with the GDPR national regulations, a pre-notification letter was emailed to potential participants providing them with the necessary information on the aims and expected outcome of the focus groups. Participants have been ensured regarding the voluntary nature of their participation in the study, the confidentiality of their answers collected during the focus groups, the privacy protection and the anonymous analysis of the collected data, and the possibility to withdraw from the study at any time and for any reason. Thus, each participant provided informed written consent and his/her permission for the recording of the focus groups. 

### 2.3. Procedures

**Figure 1:**
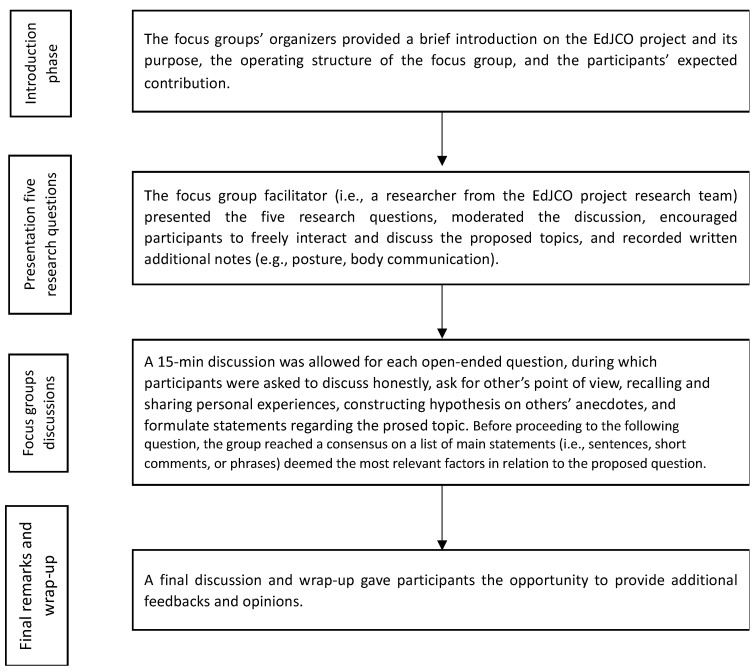
Flow chart of the focus group procedures.

Between May and October 2021, seven focus groups were organized in presence and online according to the current COVID-19 pandemic national-specific regulations in Croatia, Italy, Malta, Romania, Slovenia, Spain, and Türkiye. The common structure of the focus groups encompassed four phases, with an overall average duration of around 100 min (Figure 1). First, the focus groups’ organizers provided a brief introduction to the EdJCO project and its purpose, the operating structure of the focus group, and the participants’ expected contribution. In particular, participants were required to share their perceptions of judo coaches’ educational needs when training older judo practitioners in relation to the main identified research themes. Second, the focus group facilitator (i.e., a researcher from the EdJCO project research team) presented the five research questions, moderated the discussion, encouraged participants to freely interact and discuss the proposed topics, and recorded written additional notes (e.g., posture, body communication). Third, A 15-min discussion was allowed for each open-ended question, during which participants were asked to discuss honestly, ask for others’ points of view, recall and share personal experiences, construct hypotheses on others’ anecdotes, and formulate statements regarding the prosed topic. Before proceeding to the following question, the group reached a consensus on a list of main statements (i.e., sentences, short comments, or phrases) deemed the most relevant factors in relation to the proposed question. Fourth, a final discussion and wrap-up gave participants the opportunity to provide additional feedback and opinions. As a concluding remark, participants were invited to fill out a quality assessment questionnaire and to provide further considerations by writing an e-mail to the organizers.

### 2.4. Data Analysis

The statements recorded during each national focus group in relation to the five proposed open-ended questions have been recorded in the respective seven national languages. The translation guidelines encompassed a faithful preliminary translation of each statement into English, which was independently performed by two components of the research group from each country. Then, to ensure the accuracy of the English translation, an English reviewer was involved in a blind-back translation procedure. Thus, the final list of the English version of the statements in relation to the five open-ended questions was achieved. 

The procedures to analyze the collected data have been consistent with the original purpose of the study and the need to generate eminence-based knowledge in relation to the research questions for the identification of judo coaches’ educational needs in implementing training programs for older adults [40]. To harmonise findings emerging from the different national contexts in relation to the same general topics, a meeting of the EdJCO project members (N = 18) was organized to discuss the recorded statements and to reach a consensus in relation to the five research themes and preliminary thematic analysis to structure content units (i.e., the syntax or structure in which a registered unit/statement occurred), which provided the initial framework for further data analysis (i.e., understanding and ordering the registered statements) [41]. Repetitions within and between each of the five main thematic areas have been deleted, and partial statements have been reformulated and/or summarised within broader statements. Finally, each member independently assessed the clarity of the statements included in the final list by means of a 5-point Likert scale (i.e., ranging from 1-not clear at all to 5-very clear).in particular, a collegial consensus was considered crucial to avoid potential cultural influences deriving from different European settings [42].

## 3. Results

Overall, eighty-eight experts provided their written informed consent to volunteer for this study (F = 35%; age: 40.5 ± 15.4; university degree = 100%, judo expertise = 56%). The initial list of 262 statements was synthesized, validated, analyzed, and organized into a final list of 55 statements and six macro-areas and sub-groups (Table 1). According to the meaning of the six macro-areas, 10 statements (18%) have been assigned to the aging process area, 6 statements (11%) to the safety and first aid area, 12 statements (22%) to the physiology and fitness area, 11 statements (20%) to the psychology and mental health area, 5 statements (9%) to the organization and environment area, and 11 statements (20%) to the adapted judo teaching and training, respectively. The 10 statements included in the aging process are regarded as the general knowledge of the aging process and health (numbers 1 and 2) and the age-related degeneration of systems and apparatuses (numbers 3–7) and functions (numbers 8–10). The safety and first aid content unit mainly regarded past and present medical conditions (numbers 11 and 12), nutritional habits (numbers 13 and 14), and safety measures (numbers 15 and 16). Statements related to physiology and fitness mainly concerned functional fitness and its evaluation (numbers 17–26) and two items related to general and judo-specific literacy (numbers 27 and 28). The statements associated with psychology and mental health encompassed psychological personal traits, characteristics, and disorders (numbers 29–33), self-related aspects (numbers 34 and 35), cognition and motivation (numbers 36 and 37), and concerns (numbers 38 and 39). For organization and environment, 4 statements (numbers 40–43) accounted for social relations and engagement, whereas 1 statement pertained to the organization of the judo environment (number 44). Adapted judo teaching and training included 5 aspects pertaining to the judo setting (numbers 45–49), 4 relating to judo methodology (numbers 50–53), and 2 pertinent to the participant’s evaluation (number 54) and potential disengagement (number 55), respectively.

## 4. Discussion

The present study’s novelty lies in experts’ contribution to uncovering the relevant knowledge coaches need to acquire to coach safely and effectively older judo practitioners. According to the experts, aging processes (e.g., chronic diseases, health-related aspects), first aid (e.g., emergency action plans and contacts, hygiene, and nutrition), individual motor literacy and functional fitness, assessment tools, and evaluations, psychological (e.g., self-confidence, fears), organizational (e.g., dojo, spaces) and technical (e.g., ukemi, kata) aspects emerged as the main needs of education of judo coaches. The conceptualization of essential contents for judo educational curricula is crucial for helping coaches understand the objectives of judo programs for older adults and the necessary knowledge of practitioners’ health, abilities, and potentials, and recognizing the problems older judo practitioners may encounter. This aspect is particularly relevant in considering that coaching education has almost exclusively focused on youth, adolescent, and young adult athletes [43]. Engaging experts from the scientific and judo international communities and from different cultural settings to highlight the main components of judo programs in late adulthood, this work not only implements the current evidence-based knowledge but also represents an intersection between theory and practice in the complex area of judo training and aging [10,11,44]. In fact, three of the six identified clusters are related to the bio-medical dimensions (e.g., aging process, safety and first aid, and functional fitness and evaluation, 45 statements), two to the psycho-social dimensions (e.g., psychology and mental health, and organization and environment; 28 statements), and one to the technical dimensions (e.g., teaching and training; 26 statements) of adapted judo, substantiating the need of an integrated multidisciplinary and holistic approach when coaching older individuals [25,43,45].

### 4.1. Bio-Medical Dimension

The experts highlighted the necessity to raise the awareness of judo coaches on the aging process, which is characterized by pronounced differences between individuals due to a multi-factorial model where a natural devolution of physiological and functional abilities, high exposure to chronic diseases, and reduced active lifestyles mutually influence each other, resulting in a high incidence of chronic diseases and long-term consequences on health [45,46]. According to the experts, judo coaches should be aware that individuals older than 60 years might present one or more chronic diseases frequently observed with advancing age, such as cardiovascular diseases (30%), diabetes (25%), musculoskeletal diseases (18–38%), respiratory diseases (10%), neurodegenerative (9%) and psychological disorders (7%), and mild-to-severe visual (95%), and hearing (20%) impairments [45,47,48,49,50,51,52,53,54,55,56,57]. Furthermore, the worsening of health conditions among older individuals and the use of medications for one disease could accelerate the aging effects on other systems or apparatuses [58,59,60]. Finally, the health status of older judo practitioners should be considered not only in terms of the severity of symptoms but also in relation to the effects on physical, mental, and cognitive capabilities. 

Although exercise training is a recommended non-pharmacological treatment for individuals with hypertension and other coronary diseases, exercise modality elicits different effects during and after the training session, also in relation to the training status of the practitioner [61,62,63,64,65]. In fact, regular training could foster submaximal work tolerance by reducing at a given submaximal intensity the cardiac work and oxygen demands from the heart and the muscles [61,66,67]. However, during training sessions, coaches should consider the effects of arterial blood pressure variability due to clinostatic/orthostatic postures and isometric contractions when performing grappling techniques [68]. In this study, experts also highlighted the risks of isometric grappling techniques for practitioners with hypertension, bypass, and pacemaker. To avoid novice judo practitioners engaging in isometric contractions of the upper body when over-gripping the judogi (typical clothes of judo practitioners) of the opponent, coaches should apply a gradual-based approach to correct techniques, emphasizing the collaboration with the partner and the correct breathing during training [23]. In considering the incidence of diabetes among aged individuals, coaches should be aware that this non-communicable disease might affect the metabolic function of muscles, as well as the visual and vestibular components of balance, determining an increased exposure to falls and a worsening of the quality of life of older individuals. Furthermore, foot pain and the use of psychotropic medications and polypharmacy in diabetics increase the risks of falls. In adopting a multi-component adapted judo approach for older individuals, coaches should combine aerobic and resistance exercises, which ameliorate insulin sensibility, balance receptors, lower-limb strength, gait performance, and falling skills [10,16,69,70]. With advancing age, also muscular (sarcopenia) and skeletal (osteoporosis) degenerations are major causes of falls and fall-related injuries, often causing the loss of independence [71]. Conversely, break-down judo falling techniques (ukemi) have shown positive results in reducing bone impact force and velocity with respect to ‘natural’ fall, and judo proved to be an effective exercise modality to provide osteogenic stimuli and maintain bone health [16]. In being a situational and multi-modal exercise requiring sensorimotor control to perform appropriate techniques with accuracy and timing in relation to an opponent, judo also enhances the performance of higher-level cognitive processes (e.g., attention, working and long-term memory, processing speed, and inhibition), which prevent neurodegenerative disorders [72]. In considering the high incidence of mild-to-severe visual and hearing impairments with advancing age, to help older individuals clearly understand the coach’s technical inputs, it is necessary to ensure visible access to the coach, who has to adopt clear verbal communication or practical examples, encompassing exercises performed in a couple. In fact, reduced coordination and balance are also due to problems occurring in the inner ear, whereas static and dynamic balance training, dynamic visual acuity, and muscle strength can help hearing-impaired judo practitioners ameliorate their balance and coordinative skills [73,74]. Particular attention to exercise modalities should also be assumed when older practitioners present acute or chronic respiratory infections, pulmonary diseases, or survived lung cancer [75,76]. To foster the human respiratory system while training in judo, a spacious and ventilated indoor environment should be used, with an ambient temperature of 20 degrees Celsius [77]. To avoid the transmission of diseases, it is suggested to clean judo mats and other reusable equipment after every session [78]. 

In relation to safety and first aid aspects, during the focus groups, the experts specifically addressed the necessity to require a family medicine or a sports medicine certification that includes general health, cardio-pulmonary, metabolic, and musculoskeletal assessments. In countries where sports medicine certification is not mandatory, judo coaches could administer AAHPERD Council on Aging and Adult Development Medical/Exercise Assessment for Older Adults [79], which encompasses demographic information, activity level during the past year, and health history (presence of symptoms, smoking, and alcohol consumption, existing health concerns, medications and of dietary supplements regularly taken), to be filled in by both participants and the physician (for the physical examination section). Furthermore, recommended guidelines on practicing judo safely address general standards for first aid, emergency action plans and contacts, hygiene, and nutrition [80].

In this study, experts urged coaches to be aware of age-related dehydration, which could be due to physiological (e.g., decreased sensitivity to thirst, kidney function, medications affecting body water) and behavioral (e.g., fear of incontinence) factors. In fact, dehydration could negatively impact performance, cognitive functions, bronchoconstriction and related pathogenesis of certain pulmonary disorders, and vision [81,82,83,84]. Therefore, coaches should be urged to promote water intake during the training sessions to avoid further exercise-related dehydration [85]. Concerns related to coaches’ awareness of age-related nutritional deficiency emerged, and the role of judo practice to educate older judo practitioners on the positive effect of a balanced diet for preventing protein and micronutrient (e.g., vitamin 12 and vitamin D) deficiencies [86,87].

A thorough knowledge of the health status, previous injuries, and physical activity experience of every novice practitioner is crucial to adapt the judo training and techniques to the individual characteristics of the older judo practitioners and to ensure well-controlled interventions [11,22,44]. In particular, previous physical activity experiences could have contributed to individual motor literacy, which represents a basis for further judo capabilities. The experts highlighted the necessity for coaches to be able to evaluate the functional fitness (flexibility, coordination, strength) of the practitioners by means of field assessment tools. Thus, increasing interest has been given to the evaluation of the perceived health of older individuals as well as to the assessment of their functional fitness levels (i.e., the physical capacity to perform normal daily activities safely and independently without undue fatigue), which is crucial to maintain quality of life with advancing age [4,10,16,88,89]. In the literature, field-based functional fitness evaluations include anthropometric characteristics (e.g., height, body mass, and waist and hip circumferences), performance variables of endurance, flexibility, lower and upper body strength, coordination, and static and dynamic postural control of older individuals and older judo practitioners [10,16,79,90,91,92,93,94]. Because fitness evaluations could be time-consuming, coaches should include field-based measurements during the training sessions, also involving the practitioners in data collection when possible. To monitor the perceived intensity and enjoyment of the training, the rating of perceived exertion (RPE), the visual-analog scales (VAS) for enjoyment, and the fear of falling scale (FoF) could be easily administered at the end of training sessions [10,95,96,97,98,99]. Finally, the practitioners could rate their hydration state by means of the 8-point urine color chart, which is helpful in preventing exercise-related dehydration [100]. 

### 4.2. Psycho-Social Dimension

In this study, the experts emphasized the necessity of adopting strategies to increase older judo practitioners’ self-confidence through self-development-based goals and an enjoyable environment in the dojo, which could increase their mental and psychological health. In fact, multimodal physical activity interventions over long periods, high functional fitness, and activity engagement are considered effective preventive measures for mental disorders affecting more than 20% of middle-aged and older adults presenting relevant disturbances in thinking, emotional regulation, anxiety, behavior control, dementia, depression, and Alzheimer’s diseases [50,52,56,101,102,103]. Whilst group judo training could represent an appropriate intervention that provides rewards and reinforcement for adopting healthy active lifestyles, diet, and sleep behaviors, inactivity, and social disengagement with advancing age might be caused by feelings of social awkwardness when setting performance-based physical goals (e.g., competitiveness) rather than self-development-based goals (e.g., mindfulness) and potential achievements relevant to everyday life [104,105,106]. Thus, coaches should plan enjoyable training sessions, including skills that make older judo practitioners feel competent and willing to continue improving [11,25]. In case judo practitioners lose their self-confidence with advancing age, coaches could still play a fundamental role in helping them restore their self-efficacy by focusing on revisited goal settings [25]. To engage older judo practitioners in setting their training plan, coaches should have deep judo knowledge to explain the reasons behind the proposed exercises and have to adopt simple instructions ensuring that the older judo practitioners can easily follow them and avoid frustration [11,25]. Furthermore, the experts also raised some concerns regarding dementia and the prevention of its potentially harmful development (e.g., Alzheimer’s disease), highlighting the role played by a balanced resting blood pressure to be restored at the end of the judo training session by administering relaxation exercises and classical music therapy [107,108].

The experts claimed that fears (e.g., fear of falling, training barefoot, contact with partners, new ground-grappling techniques, serious injuries) and anxiety disorders could be potential obstacles for older practitioners to engage in judo training, and suggested that a practitioner-based approach rather than traditional and/or conventional training protocols should be adopted [105,109,110]. In fact, over time, adapted judo training has been effective in reducing fears of falling among older adults [11,111,112,113,114]. 

Regarding organizational and environmental aspects, during the focus groups, the expert emphasized the role of the family, friends, and group relationships in supporting the engagement in judo of older practitioners, as well as in decreasing the social isolation, loneliness, and depression, which increase with advancing age [105,115]. To deepen social relations while training and increase engagement in daily physical activity, the experts suggested that a judo program for older adults should be focused on the enjoyment of the relationships and cooperation with other judo practitioners and the personal improvements rather than on the communication of the benefits of physical activity. In this respect, the intrinsic values of judo could be used as a means to unify age-related communities and to foster family relationships breaking barriers between different generations when embracing an intergenerational approach with children and grandchildren [115]. Finally, to foster positive feelings, social interaction, and an open atmosphere, experts highlighted the role of background music (music genres suggested included oldies, pop, nursery rhymes, and world-famous songs) while training judo. While music-driven or music therapy exercise is well known for its positive nonpharmacological effects [116], background songs could have an ergogenic effect in decreasing negative feelings and stress, perceived exertion, as well as in enhancing social interaction, enjoyment, physical performance, and physiological efficiency among older practitioners [117,118].

### 4.3. Technical Dimension

According to the expert’s opinions, coaches have to apply specific strategies and methodologies to achieve positive outcomes (e.g., prevention of fall-related injuries, promotion of wellness and active lifestyle, sense of belonging, and lifelong learning) from an adapted judo training [11,20,22,111]. A judo training methodology for older adults respecting the general good principles of safety, customizability, progression, completeness, enjoyment, and variety was supported. A fruitful discussion addressed specific aspects assuring the necessary flexibility to respect the personal experiences, creativity, and autonomy of coaches in planning their training according to the needs, safety, state of health, physical fitness, and degree of ability of the class of seniors. In particular, for individuals older than 60 years, techniques used to grasp the opponent’s joint (kansetsu-waza) and techniques of strangulation (shime-waza) should be avoided or practiced on the kata modality only, whereas break-fall judo techniques (ukemi-waza) and techniques from a standing position (tachi-waza) and on the ground (ne-waza) are recommended [11]. The highlighted main principles encompassed a practitioner-based approach, with adapted judo sequences initially simplified and gradually increasing in difficulty by favoring first static or slow exercises practiced individually to smooth dynamic performances executed in pairs, and finally changing partners. In considering the adaptable nature of judo, coaches could select a variety of exercises suitable for the peculiarity of the older judo practitioners, always in the spirit and logic of the judo methodology. The experts underlined that the best exercise plan is not just what older individuals feel comfortable performing but what older judo practitioners enjoy and are likely to practice on a regular basis. Although older adults are recommended to practice 45–50 min physical activity every day [4], at the beginning of a judo program, coaches should engage them in at least 2–3 h of moderate-intensity judo per week, ideally spread over several days. In fact, aging-related limitations and low perceived value for ameliorated quality of life are known barriers to older adults’ physical activity engagement [119]. In considering those realistic goal settings are crucial to ensure adherence to the program and to prevent dropouts, the participants’ feedback and a friendly and empathic atmosphere were presented as important aspects of the coach–practitioner relationships and coach competence trust [25,120]. Another issue pertains to the organization of judo group sessions, and judo coaches are urged to consider the age, sex, anthropometry, health status, expertise, and fitness level of the older judo practitioners before defining their training plans. 

It is well known that falls account for the second leading cause of unintentional injury and deaths worldwide [109]. Training basic judo breakfall skills and applying judo knowledge proved to have positive effects on the fear of falls among older people, which could decrease the incidence of fall-related injuries [44,111,113]. To avoid injuries from falling, Kano Sensei introduced a first static approach to break-falls (Ukemi) for efficiently controlling the falls produced by judo throwing techniques. Despite this first step of knowledge, individuals might not be prepared to control falls while moving, which are the most frequent during daily life situations causing injuries [110]. According to the experts, an optimal solution would be to use simplified sequences of codified movements (e.g., forms; kata) that use the fundamental principles and opportunities to teach falling control during dynamic situations (ukemi). Breakfall techniques have to be adjusted to the capabilities and age of the practitioners, including multimodal exercises useful for learning roll-related movements [121]. Despite the most dangerous falls occurring in the forward direction, the forward falls in judo (mae-mawari ukemi) are not recommended for older adults because they are too complex, requiring advanced preparedness of the judo practitioner and a specific coaching methodology [122].

Experts also highlighted the necessity that coaches should guide practitioners with clear and simple communication to avoid deleterious misunderstandings that can generate incorrect or dangerous executions. Furthermore, to facilitate the learning of judo techniques while preserving the safety of the older practitioners, coaches are encouraged to use mats, exercise balls, balance boards, and resistance bands, also using background music, which could correspond to the requirements for the organization of learning and training processes in judo [123]. Another aspect that emerged as important was the necessity to pose particular attention to a well-structured warm-up, correct breathing patterns when exercising, and frequent breaks and relaxing cool-down following intense sequences.

### 4.4. Limitations and Future Directions

The present work was the first EU-funded collaborative partnership addressing the issues of educating coaches for older judo practitioners. In complementing the evidence-based knowledge on judo training in older practitioners, the present participatory approach provides multidisciplinary experts’ experiences and opinions to be considered in the construction of an educational program for judo coaches of older adults. To improve the usability and satisfaction of a sound educational program for the implementation of competencies of judo coaches with respect to older practitioners, a major limitation is still present. In fact, there is a need for further investigations to provide empirical validation of relevant necessary competencies coaches should possess when training older judo practitioners. Actually, structured and comprehensive education for coaches of individuals with advancing age is still limited [25]. Therefore, future works should gather information from the end-users within and beyond Europe regarding their actual knowledge of training older individuals and the relevance of the aspects highlighted by the experts during the focus groups. In adopting this ethnographic research approach through a large-scale survey, high-level judo coaches’ perceptions on the relevance of the aspects emerging from the present study and their actual need for training could help the construction of a sound educational program for coaching judo in late adulthood. 

## 5. Conclusions

The present international eminence-based study investigated the expert’s perspectives regarding the necessity of a tailored education for judo coaches of older practitioners, harmonizing diverse intercultural perspectives. The experts addressed the major needs of coaches’ education, including the most relevant age-related chronic diseases, psychological (e.g., self-constructs and fears) and health (e.g., hydration, nutrition, sleep) aspects that characterize the aging process, the needs of risk prevention and emergency action plans, the adapted techniques to meet the functional fitness and motor literacy of older practitioners, the evaluation tools, and the organisational (e.g., dojo, spaces) arrangements. In fact, the role of judo coaches is crucial when addressing the multifaced challenges of providing adequate training programs to a heterogenous older adult population (e.g., former master athletes, novices, and different sports backgrounds). Thus, to apply a practitioner-based approach and promote active, healthy aging, a trustful and empathic relationship between judo coaches and practitioners is a milestone.

To propose judo coaches as a user-friendly tool to facilitate a sound practitioner’s self-development program, our work introduces novel guidelines to promote judo lifelong learning for older people. Due to falls-related injuries, older people are facing disabilities and psychological diseases (e.g., depression, insecurity, poor self-confidence). In this respect, judo could prevent fall-related injuries by engaging older people in a person-centered approach to learning judo breakfall techniques [122]. Thus, judo coaches need a specific adult-oriented approach to enhance the quality of older adults’ sports experiences by linking safety, enjoyment, social interactions, and learning principles. Future research on judo training for older practitioners is needed to substantiate the knowledge that this sport discipline might prevent falls with advancing age [111,122,124].

## Figures and Tables

**Table 1:** List of macro areas, sub-groups, and statements to develop an educational judo program for coaches of older practitioners.

	**Macro-Areas**					
	**Aging Process**	**Safety and First Aid**	**Physiology and Fitness**	**Psychology and Mental Health**	**Organization and Environment**	**Teaching and Training**
**Sub-groups**	Immune function Metabolic health Musculoskeletal health Eyes health Hearing health Healthy sleep Cardiovascular health	Medical history Medical certificate Diet and hydration Risk prevention	Evaluation Motor literacy knowledge Development of functional fitness and physical capability	Psychological disorders Psychological traits and state Attitudes and motivations of older practitioners to practice judo Activation and relaxation status Body image Individual and group empathy Mood and emotional status Fear	Economic status Family and social support, relations, and engagement Living conditions (e.g., autonomy and independence) Spaces (e.g., dojo, changing rooms, lighting, etc.)	Adapted falling techniques for older practitioners (e.g., falling techniques) Variability of practice (e.g., introducing different sports to judo practice) Realistic goals through acute and long-term effects of judo training Training methodology and monitoring (e.g., workload, warmup and cooldown, rating of perceived exertion) Proactive participation and engagement (e.g., attendance) Group division or inclusion (e.g., age, athletic experience and capabilities, gender) Communication (e.g., efficacy and efficiency) Friendly context (e.g., openness, enjoyability)
**Statements**	1. General knowledge of aging process	11. Medical history	17. Functional fitness	29. Psychological traits and states	40. Social values and relations	45. Realistic and sustainable goal setting
	2. Aging health	12. Current health condition	18. Development and evaluation of body mass	30. Empathy and personal values	41. Living conditions (e.g., family, friends, independence, etc.)	46. Clear and effective communication and guidance
	3. Musculoskeletal aging	13. Diet and hydration	19. Development and evaluation of flexibility	31. Mood and emotional status	42. Collaboration and team playing	47. Positive atmosphere favoring sense of belonging, engagement, and training adherence
	4. Cardiovascular aging	14. Alcohol intake	20. Development and evaluation of balance	32. Activation and relaxation	43. Active and sedentary behaviors	48. Adapted training tools (e.g., mats, exercise balls, balance boards, resistance bands, use of music)
	5. Immune system aging	15. Basic first aid	21. Development and evaluation of coordination	33. Psychological disorders	44. Judo physical environment (e.g., dojo, common spaces, changing room)	49. Group organization (age, sex, anthropometry, health status, expertise, fitness level)
	6. Digestive system in aging	16. Emergency measures	22. Development and evaluation of upper and lower body strength	34. Self-constructs (e.g., control, efficacy, confidence)		50. Basic training principles (safety, graduality, smooth progression variety, enjoyability)
	7. Neurological aging		23. Development and evaluation of endurance	35. Body image		51. Adapted training load (frequency, duration, intensity, rest-exercise ratio)
	8. Sensory system aging		24. Development and evaluation of agility	36. Exercise and cognition (e.g., attention, focus, shifting, memory, reasoning, anticipation, etc.)		52. Importance of prolonged warm-up, frequent breaks, breathing patterns, and relaxing cool down
	9. Aging metabolism		25. Development and evaluation of judo skills	37. Attitudes and motivation to practice and learn judo		53. Adapted judo techniques to ensure safety and prevent injuries
	10. Healthy sleep in aging		26. Field-based functional fitness tests	38. Fear of falling		54. Older practitioner’s feedback
			27. Motor literacy (i.e., learning and control)	39. Judo-related uneasiness and fears (e.g., contact with partners, training barefoot, new exercises, ground fighting, etc.)		55. Burnout and dropout prevention
			28. Judo-specific literacy			

## Data Availability

The data presented in this study are available on request from the corresponding author.

## Supplementary Materials

The following supporting information can be downloaded at: https://www.mdpi.com/article/10.3390/sports11080143/s1, Supplementary Material S1. Guidelines for the Development of the EDJCO WP2 National Focus Groups with Scholars/Coaches/Judo experts.
