# Supplementary material for: Educational Needs for Coaching Judo in Older Adults: The EdJCO Focus Groups
Source: Sports (Basel). 2023 Jul 31;11(8):143. doi: 10.3390/sports11080143 (PMC10458867; doi:10.3390/sports11080143)
Supplement: Supplementary file 1 [file sports-11-00143-s001.zip › Supplementary Material S1. Guidelines for the Development of the EDJCO WP2 National Focus Groups with Scholars-Coaches-Judo experts.pdf]

Supplementary Material S1

622155-EPP-1-2020-1-IT-SPO-SCP

Guidelines for the Development of the EDJCO WP2 National Focus Groups with Scholars/Coaches/Judo experts.

Aim

To conduct a research study to develop a framework on the relevant factors to be addressed in a Judo Education Model for Coaches of Older Practitioners based on experts' opinion within the EDJCO Partners and amongst the wider community of stakeholders (e.g., former athletes, coaches, expert in geriatrics, physiology, sports medicine, orthopaedics, psychology, etc.). A comprehensive analysis of the eminence-base knowledge of scientific experts in adapted physical activity for older individuals and judo coaches. The outcomes will be summarized in materials for a scientific manuscript.

Rationale

Appropriate representativeness of the focus groups in terms composition (Parker & Tritter, 2006), and a purposeful sampling will deem the core strength for a comprehensive practical knowledge through the provision of the experts' opinions regarding the needs, challenges, risks, and benefits of judo training for older individuals. To ensure a robust and authentic eminence knowledge and new insights on the relationships between relevant factors to guide the educational methodology to be developed in WP3, the recruitment of at least 10 participants meeting the following inclusion criteria: scholars (university professors and researchers expert in biomechanics, communication, education, kinesiology, nutrition, physiology, and psychology), medical doctors (with a specialization in geriatrics, family medicine, and sports medicine), and judo (>second DAN with a coaching expertise >5 yrs) and other combat sports experts. Participants will be accessed and invited (APPENDIX S1) through the Partner's networks through a fine expertise-based stratification.

Participation in the Focus Groups will be based on a process of informed consent designed to lead to a thorough understanding of the research project. At the end of the focus groups, participants will be asked to establish in an open-ended and non-judgmental fashion an exhaustive list of all potentially relevant factors for participation to judo in older ages.

The information collected in the languages of the national focus groups will be translated into English and back translated into the original language by the

Partners. The organizers of the focus groups must send the WP2 Leaders the excel file with the list of statements collected during their workshop. The WP2 Leaders will collect and analyse the comprehensive summaries of the focus groups and will provide a preliminary list of identified relevant factors related to the educational needs of judo coaches to be circulated and approved by the Partners. This list of factors will be further informed by the outputs of the scientific literature.

Methodology to develop the stakeholders' contribution during the focus group on relevant issues to be considered in the EDJCO Education Programme
The national workshop with experts with the **aim to collect information on relevant issues to be considered in the Education Programme for Coaches of Older Judo Practitioners**.

Questions to be addressed to the participants:

Based on your experience(s), discuss and list:
1. Specific to your field of expertise, what the most relevant benefits judo coaches should be aware when they train former or novice older judo practitioners?
2. Specific to your field of expertise, what the most relevant information judo coaches should be aware when they train novice older judo practitioners?
3. Specific to your field of expertise, what the most relevant information judo coaches should be aware to prevent/manage risks when they train both or former or novice older judo practitioners?
4. Specific to your field of expertise, which are the main criteria in defining judo training groups in relation to participants' judo expertise, chronological/functional age and/or sex?
5. Specific to your field of expertise, what the most relevant tools/tests/measurements for monitoring training plans for former or novice older judo practitioners?

The 4 phases of the workshop

1) a short (5-10 min) presentation of the EDJCO project (e.g., its aims and the expected contribution of participants and organization)
2) a 15-min discussion is allowed for each of the five questions during which participant were asked to discuss ask for other's point of view, recalling and sharing personal experiences, constructing hypothesis on others' anecdotes, and formulate statements regarding the prosed topic.
3) Before proceeding to the following question, the group reached a consensus on a list of the main statements (i.e., sentences, short comments, or phrases) deemed the most relevant factors in relation to the proposed question.
4) A final 15-min general discussion and wrap-up to give participants the opportunity to provide additional feedbacks and opinions. Then, the organizer will provide a general sum-up, and thanks the participants.

Reports of the National Focus Groups

The organizers of the focus group must **report to the WP2 leaders**:

1) the list of participants.
2) a brief report of the workshop including the frequency of occurrence in percentages of the participants' answers to the quality questionnaire.
3) the list of factors arising from the discussion in a excel file. Figure S1 provides an example of the report intended only as a starting point.

Figure S1. Example of a report from a focus group

benefits judo coaches should be aware	information judo coaches should be aware	prevention/managing risks	defining judo training groups	tools/tests/measurements for monitoring training plans
xxxxxxxxxxxxxxx	xxxxxxxxxxxxxxx	xxxxxxxxxxxxxxx	xxxxxxxxxxxxxxx	xxxxxxxxxxxxxxx
yyyyyyyyyyyyyyy	yyyyyyyyyyyyyyy	yyyyyyyyyyyyyyy	yyyyyyyyyyyyyyy	yyyyyyyyyyyyyyy

Appendix S1 – Invitation Letter and Consent Form

Dear Expert,

On behalf of the Consortium involved in the European project "EDJCO" (www.EdJCO.eu), your expertise and knowledge in supporting a judo coach managing his/her training session for older judoka have been considered very valuable in assisting us during a focus group to develop an Education Programme for coaches of older judo practitioners.
We ask that you read this form and ask any questions that you may have before agreeing to be in the study, which received the ethical approval from the University of Rome Foro Italico (CAR48/2020/INT/2022).

The main aim of the focus group is to identify the most relevant factors potentially important in providing valuable information for coaches of older judo practitioners. To this purpose, the project will contribute to the lifelong education of judo coaches and active lifestyles with advancing age. In particular, the project intends to establish an on-line multi-lingual educational programme for judo coaches. The educational programme will be based on evidence- and eminence-base knowledge of experts.

If you agree to be in this study, you will be asked to participate in a focus group and will be asked to discuss the following questions:

1. Specific to your field of expertise, what the most relevant benefits judo coaches should be aware when they train former or novice older judo practitioners?
2. Specific to your field of expertise, what the most relevant information judo coaches should be aware when they train novice older judo practitioners?
3. Specific to your field of expertise, what the most relevant information judo coaches should be aware to prevent/manage risks when they train both or former or novice older judo practitioners?
4. Specific to your field of expertise, which are the main criteria in defining judo training groups in relation to participants' judo expertise, chronological/functional age and/or sex?
5. Specific to your field of expertise, what the most relevant tools/tests/measurements for monitoring training plans for former or novice older judo practitioners?

We ensure you that there is no right or wrong answer and no one is going to judge your opinion. The records of this study will be kept strictly confidential. We will not include any information in any report we may publish that would make it possible to identify you.

The decision to participate in this study is entirely up to you. You may refuse to take part in the study at any time without affecting your relationship with the

investigators of this study. You have the right not to answer any single question, as well as to withdraw completely from the interview at any point during the process.

You have the right to ask questions about this research study and to have those questions answered before, during or after the research. If you have any further questions about the study, at any time feel free to contact [*name of the organizer*] at [*email of the organizer*] or by telephone at [*phone number of the organizer*].

Your time and effort in participating in the focus group would be greatly appreciated and we will acknowledge your contribution in future publications related to the development of the educational programme and in the EDJCO official website.

With best regards,

NAME and SIGNATURE of the organizer
On behalf of the EDJCO Framework Development Taskforce

Consent

Your signature below indicates that you have decided to volunteer as a research participant for this study, and that you have read and understood the information provided above. You will be given a signed and dated copy of this form to keep, along with any other printed materials deemed necessary by the study investigators.

Participant's Name (Print)

Date and signature
